# New Dental Depot

**Published:** 1854-01

**Authors:** 


### NEW DENTAL DEPOT.

The Profession will be glad to learn that increased facilities for the procurement of teeth and dental materials are afforded them. S. Wardle & Co. have been gradually extending their business in this line for the past year, and they are now offering the Profession a good supply of the various articles needed in practice. They are constantly manufacturing a large supply of teeth of the various kinds, such as gum, common, plate and pivot, of the different patterns shades of color, &c. These teeth, we are glad to say, for strength, endurance of heat and natural appearance, are meeting with approbation. Mr. Wardle is a practical Block workman, and is ever ready to meet the wants of the Profession in this department, and has supplied us with several blocks of teeth, which answer an admirable purpose.

The Profession are necessarily interested in every thing which increases the supply of articles needed. The demand has become so great that any one establishment could scarcely supply the market. In justice to Dr. J. M. Brown, we would say, that we are fully aware of the labor and pains he has taken to meet this demand, and so far as the manufacturers of teeth and instruments could be brought to co-operate, this has been effected. Those of us who have been resident of the city for the past ten years, can appreciate his many efforts to serve the Profession.

It will be seen, also, that Mr. Armstrong, of Philadelphia, has established an agency for the sale of his teeth, in this city. These teeth are spoken of very highly by some of the Profession, and certainly present a very good appearance. The Profession will find Mr. Palmer very attentive and obliging, and punctual in filling all orders.

